# Slope aspect affects the soil microbial communities in karst tiankeng negative landforms

**DOI:** 10.1186/s12862-022-01986-y

**Published:** 2022-04-30

**Authors:** Cong Jiang, Sufeng Zhu, Jie Feng, Wei Shui

**Affiliations:** 1 College of Urban and Environmental Sciences, Peking University, Beijing, 100871 China; 2 Chinese Research Academy of Environmental Sciences, Beijing, 100020 China; 3 School of environmental and safety engineering, Fuzhou University, Fuzhou, 350116 China

**Keywords:** Karst tiankeng, Slope, Microbial community, Heterogeneity, Metagenomics

## Abstract

**Background:**

Karst tiankeng is a large-scale negative surface terrain, and slope aspects affect the soil conditions, vegetation and microbial flora in the tiankeng. However, the influence of the slope aspect on the soil microbial community in tiankeng has not been elucidated.

**Methods:**

In this study, metagenomic sequencing technology was used to analyze the soil microbial community structure and functional potentials on the shady and sunny slopes of karst tiankeng.

**Results:**

The Shannon–Wiener diversity of microbial communities on shady slope (SHS) was significantly higher than that on sunny slope (SUS). Although the composition of dominant phyla on shady slope (SHS) and sunny slope (SUS) was similar, there were significant differences in beta-diversity. The linear discriminate analysis (LDA) results showed that biomarkers mainly belongs to *Actinobacteria*, *Chloroflexi* and *Proteobacteria*. Functional pathways and CAZy (Carbohydrate-Active Enzymes) genes also had a remarkable response to slope aspect change. LEfSe results indicated several biomarker pathways in sunny slope involved in human disease. Moreover, the abundance of CAZy genes was higher in shady slope and had stronger ability in decomposing litter. The microbial communities were mainly correlation with the vegetation characteristics (species richness and coverage) and soil properties (SOC and pH).

**Conclusions:**

These results indicate slope aspect has a pronounced influence on microbial community composition, structure and function at karst tiankeng. In the future, the conservation of karst tiankeng biodiversity should pay more attention to topographical factors.

**Supplementary Information:**

The online version contains supplementary material available at 10.1186/s12862-022-01986-y.

## Background

The karst tiankeng is a special and grand negative landform first discovered in the karst area of southern China at the end of the twentieth century [1]. Karst tiankeng are defined as a kind of extremely large karst negative terrain with huge volume, steep and enclosed rock walls, developed in soluble rock formations (mainly carbonate rocks), with plane width and depth usually exceeding 100 m, and connected with underground rivers at the bottom [2]. Affected by the steep rock wall and its own depth, a local microclimate different from the surface of the tiankeng has been formed inside the tiankeng, which has nurtured unique resources of animals, plants and microorganisms [3–6]. As we all know, human activities have caused environmental problems such as rocky desertification, loss of land resources, and ecosystem degradation in karst areas, turning them into vulnerable and sensitive areas of the ecological environment [7, 8]. The unique ecological environment of tiankeng makes it like an oasis in a degraded karst landscape [6].

China is the kingdom of karst tiankengs, which contains more than 70% of the world’s total tiankengs, and has preserved a more systematic and complete chain of tiankeng evolution [9]. At present, most research focuses on the formation and evolution mechanism of tiankeng [10], plant diversity [4, 11], and tourism resource value [12]. However, there are few studies on the distribution of microbial resources and their mechanism of action in the karst tiankeng ecosystem. Pu et al. [5] and Jiang et al. [13] results showed that the distribution of microbial communities in the karst tiankeng ecosystem has obvious heterogeneity. As the most diverse and species-rich taxa on earth, soil microorganisms promote the energy flow and material transformation of the ecosystem and maintain the normal operation of the ecosystem [14, 15]. Previous studies have proved that soil microorganisms play an important role in karst ecosystem, especially in the process of plant community restoration, which often determines the restoration effect of different vegetation [16]. In addition, the CO_2_ produced by microbial metabolic activities will affect the carbonate karst erosion rate, and ultimately affect the process of the entire karstification [17]. Therefore, investigating the structure and function of the soil microbial community is very necessary for exploring the generation of biological diversity in tiankeng and the prediction of the evolution direction of ecosystem functions.

As an important topographic factor, slope aspect can alter solar radiation, temperature, precipitation and soil texture, thereby affecting soil nutrients and microbial communities [18, 19]. Previous studies have shown that the slope aspect has a significant impact on the microbial characteristics [20, 21]. Some studies have found that north slopes have higher microbial biomass carbon (MBC) and relative abundance of Gram-negative bacterial than south slope [20]. However, other studies have shown that the total phospholipid fatty acid, diversity of bacterial and fungal in the south slope were higher than that in north slope [22]. These results reflect the obvious influence of slope aspect on microbial community characteristics. The karst tiankeng is affected by the shelter of vertical cliffs, and the habitats of shaded and sunny slopes are distinctly different. Our previous research has found the soil properties and microclimate of shady and sunny slopes in karst degraded tiankeng were dramatically different, which results in obvious differences in the types of plant communities [23]. However, the relationships between topographic distribution of microbial communities and soil physicochemical properties in karst tiankeng were rarely investigated. Affected by the effect of karst tiankeng traps, the influence of slope aspect on the microclimate may become more important. It is vital to understand how environmental constraints affect the microbial communities in karst tiankeng. Furthermore, understanding the characteristics of the microbial community related to the slope aspect is of great significance for clarifying the role of soil microorganisms in the function of the tiankeng ecosystem. In the present study, the characteristics of microbial community between the shady and sunny slope were analyzed in the karst tiankeng. The relationship between environmental factor and microbial community were established, simultaneously. This research aims to provide reference and scientific basis for the conservation of karst tiankeng biodiversity and the understanding of soil ecological processes.

## Results

### The soil physicochemical properties on the different slope

The plant characteristics differed between the shady slope and sunny slope (Table 1). The plant species richness was significantly higher in SHS (*p* < 0.05). Significant differences in plant coverage were also found between the SHS and SUS (*p* < 0.05). TK and SWC were significantly higher in SHS (*p* < 0.05). The soil pH value was 7.12 (*p* < 0.05) higher at SUS than at SHS (6.64). (Table 2).

**Table 1:** The characterization of the plant communities feature on the different slopes

	Species richness	Coverage (%)	Shannon–Wiener index	Dominant species
SHS	42.8 ± 3.70a	76.2 ± 4.32a	2.06 ± 0.17a	*Myrsine africana* Linn *Debregeasia orientalis* C. J. Chen *Ternstroemia gymnanthera* (Wight et Arn.) Beddome
SUS	29.8 ± 5.06b	60.0 ± 5.94b	1.77 ± 0.12a	*Quercus guyavifolia* *Quercus variabilis* Bl

Values with different letters in a row denote significant difference at *p* < 0.05

**Table 2:** The characterization of the soil physicochemical properties on the different slopes

	SOC	TN	TP	TK	pH	SWC
SHS	66.48 ± 2.94a	2.84 ± 0.55a	0.45 ± 0.08a	12.48 ± 1.40a	6.64 ± 0.21b	0.44 ± 0.03a
SUS	58.95 ± 3.84a	2.43 ± 0.36a	0.49 ± 0.12a	9.22 ± 2.17b	7.12 ± 0.17a	0.37 ± 0.03b

Values with different letters in a row denote significant difference at *p* < 0.05

*SOC* soil organic carbon, *TN* total nitrogen, *TP* total phosphorus, *TK* total potassium, *SWC* soil water content

### Variation on microbial community diversity and composition on the different slopes

**Fig. 1:**
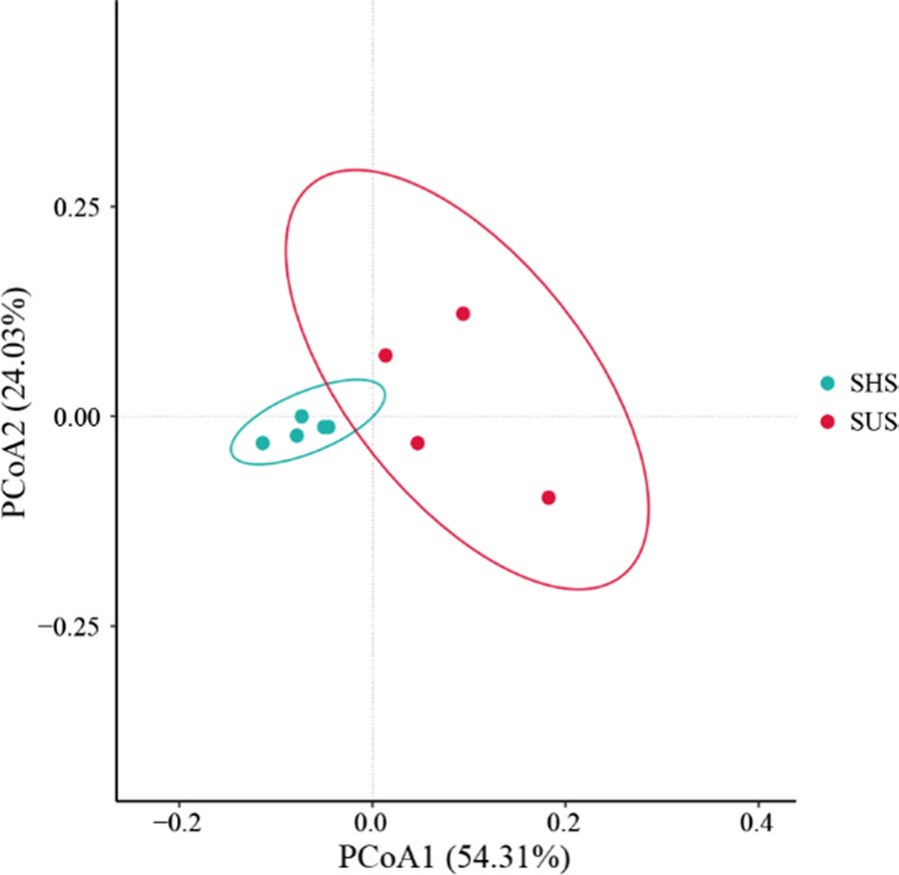
The principal coordinate analysis (PCoA) of microbial community on sunny slope (SUS) and shady slopes (SHS)

The Shannon–Wiener index was significantly higher in SHS (*p* < 0.05) (Additional file 1: Fig. S1). The ANOSIM analysis results showed that soil microbial community from shady slope and sunny slope were significantly different (R = 0.612, *p* = 0.012) (Additional file 1: Fig. S2). The PCoA results exhibited that microbial community in SHS were separately from those in SUS (Fig. 1).

According to the metagenomic analyses, the highest number of sequences corresponded to bacteria, followed by archaea, fungi and viruses. As shown in Additional file 1: Fig. S3, *Proteobacteria* was the most abundant bacteria phyla in SHS and SUS soil sample, and followed by *Actinobacteria*, *Chloroflexi*, *Acidobacteria* and *Verrucomicrobia*. In addition, twelve archaea phylum were obtained. The most abundant archaea phyla were *Euryarchaeota*, *Thaumarchaeota* and *Candidatus_Bathyarchaeota*. As an important part of soil microbes, the kingdom fungi consisted of nine phyla. Among them, the phyla *Ascomycota* and *Basidiomycota* were the most abundant.

**Fig. 2:**
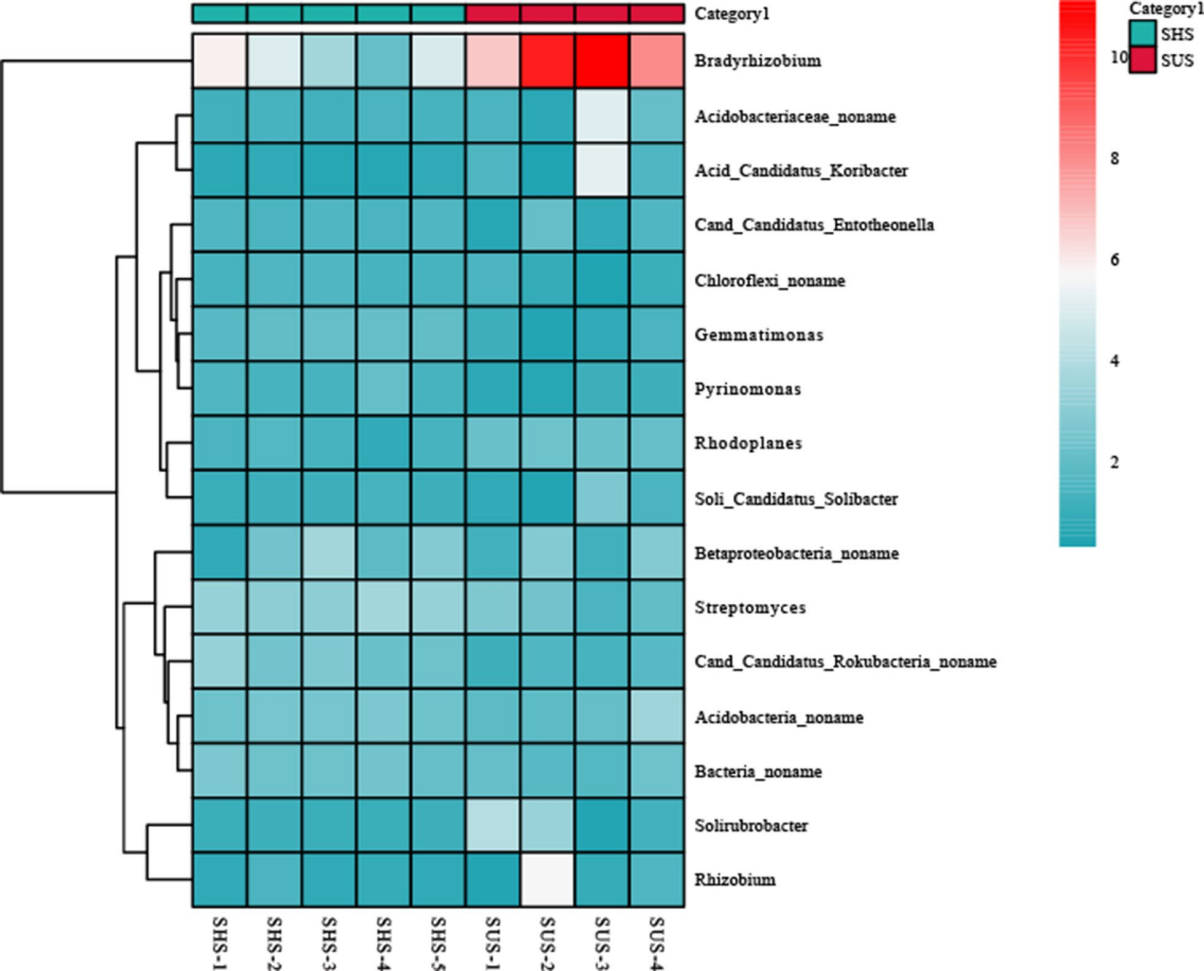
The microbial community composition of sunny slope (SUS) and shady slopes (SHS) at the genus level (The heatmap was obtained by the R software 4.1.2)

**Fig. 3:**
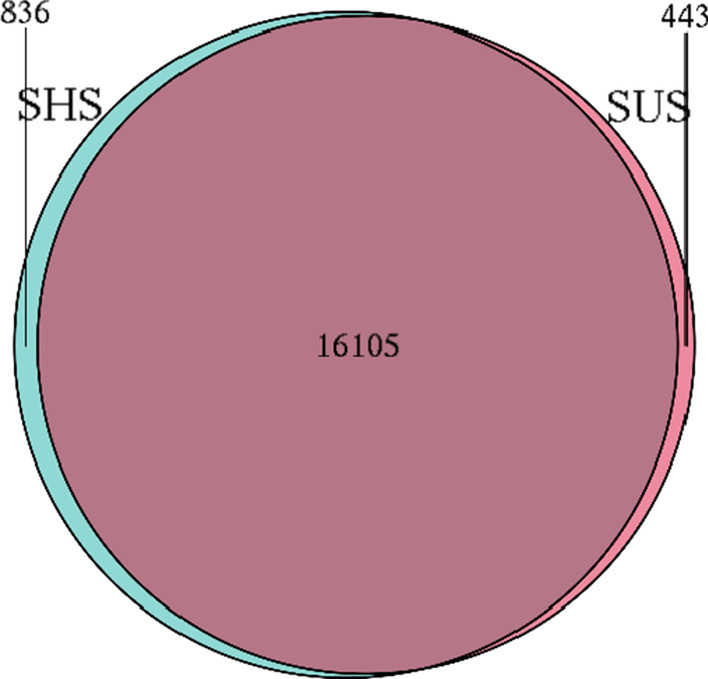
The similarity analysis of microbial community at the species level on sunny slope (SUS) and shady slopes (SHS)

The microbial community compostion at the genus level (abundance > 1%) is shown in Fig. 2. The most abundant genus in both soil samples was *Bradyrhizobium* and the abundance ranged from 4.31 to 9.04%. In addition, *Streptomyces* (2.17–3.25%), *Acidobacteria_noname* (2.32–2.47%), *Betaproteobacteria_noname* (2.06–2.31%), *Bacteria_noname* (1.99–2.35%) and *Cand_Candidatus_Rokubacteria_noname* (1.45–2.60%) were shared by both soil samples. Furthermore, Veen diagrams showed the shared and unique species between the SHS and SUS. The shared species between them was 16,105, and unique species was 836 and 443 in SHS and SUS, respectively (Fig. 3).

The LEfSe method was used to found the microbial taxa causing significant differences between the SHS and SUS. As shown in Additional file 1: Fig. S4, a total of 92 biomarkers were founded with an LDA threshold of 3.0. Most microbial clades were significantly enriched in SHS, while only 22 clades were enriched in SUS. Specifically, *Actinobacteria* (class), *Chloroflexi* (phylum), *Deltaproteobacteria* (class), *Streptomycetaceae* (family), *Candidatus_Rokubacteria_noname* (class), *Candidatus_Rokubacteria_noname* (family), *Candidatus_Rokubacteria* (phylum) were enriched in SHS. Alphaproteobacteria (class), Rhizobiales (order), Bradyrhizobiaceae (family), Bradyrhizobium (genus), Rhodospirillales (order) were enriched in SUS.

### Variation on microbial metabolic pathways on the different slopes

**Fig. 4:**
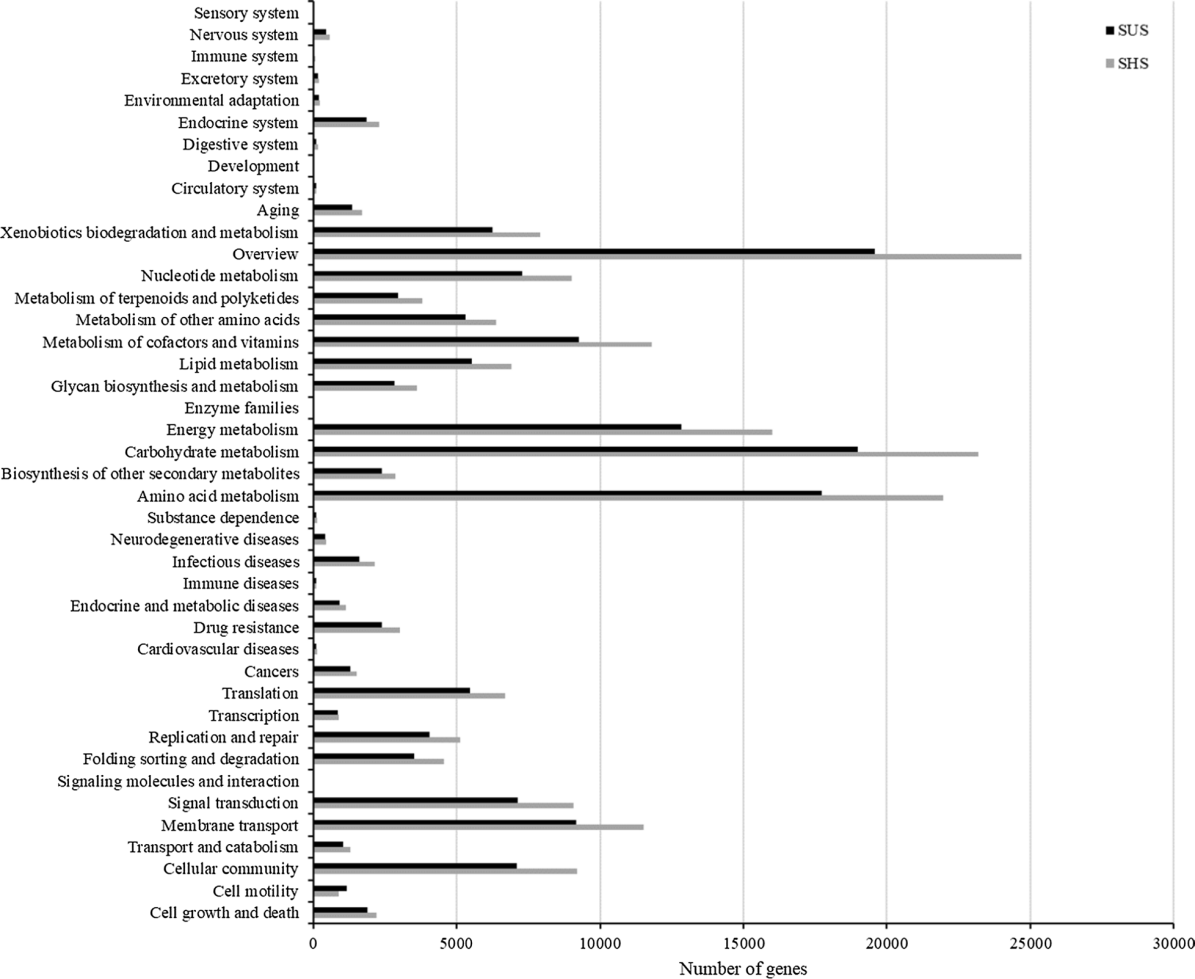
The number of genes associated with KEGG pathways on sunny slope (SUS) and shady slopes (SHS)

**Fig. 5:**
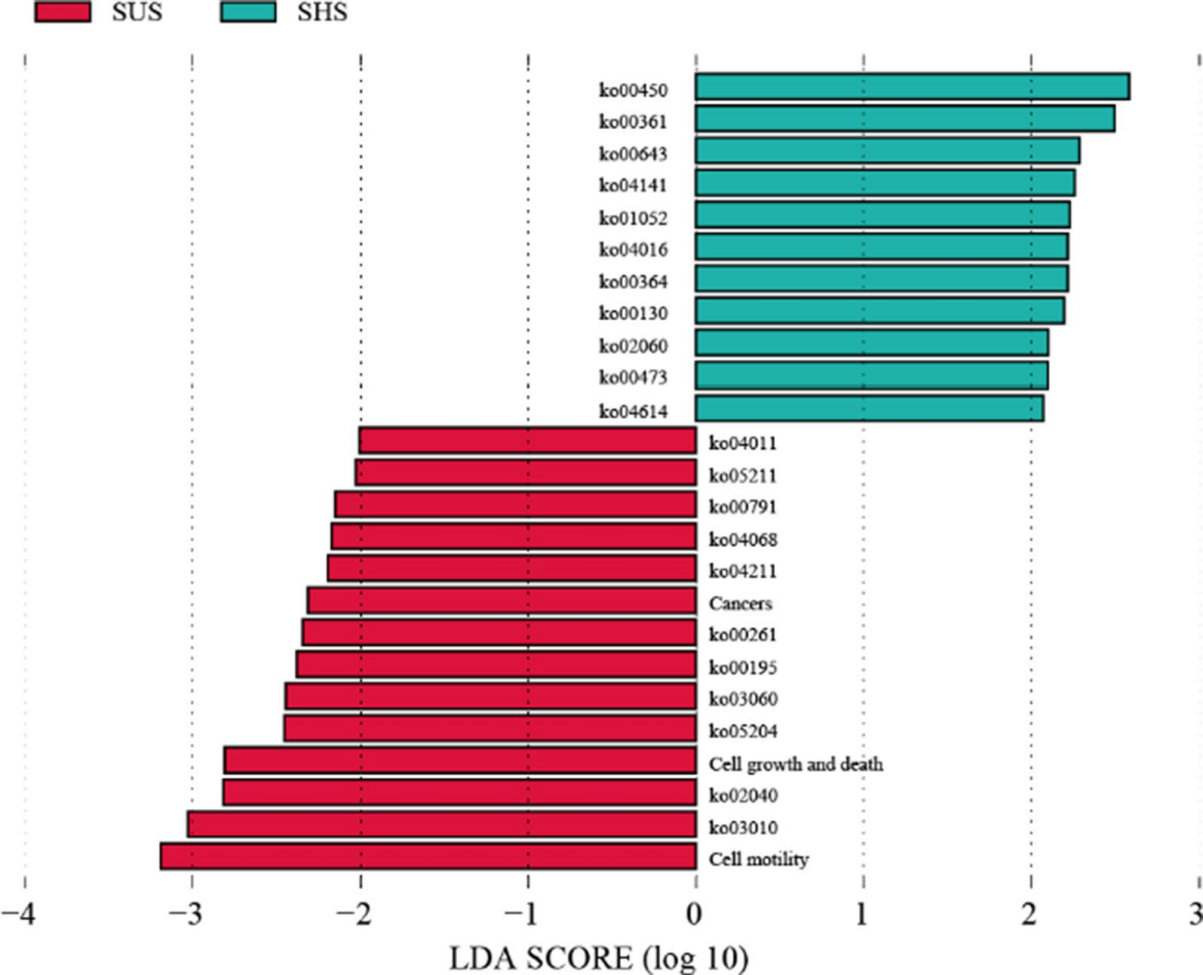
LEfSe analysis of functional categories (level 3) with significant differences (threshold value of 2.0) on sunny slope (SUS) and shady slopes (SHS)

Based on the KEGG database, the functional contributions of the microbial community in the tiankeng soil samples were annotated. A total of 4786 KEGG orthologues (KO) were annotate and mainly belonged to metabolism, environmental information processing and genetic information processing (Additional file 1: Fig. S5). Among the total of 42 functional pathways, the SHS had the higher genes number in 41 pathways (e.g., carbohydrate metabolism, amino acid metabolism, membrane transport and cellular community) (Fig. 4). Furthermore, the abundance of genes associated with C and N cycle on SUS and SHS were different. Soil microbial communities are more involved in the carbon fixation pathways in prokaryotes, pyruvate metabolism, glyoxylate and dicarboxylate metabolism and glycolysis/gluconeogenesis related to the C cycle. The genes related to the C and N cycles exhibited the higher abundances in SUS (Additional file 1: Fig. S6). The LEfSe analysis was used to detected functional pathways with significant abundance differences between the shady slope and sunny slope. As shown in Fig. 5, the microbial community in shady slope was mainly involved in membrane transport, signal transduction, folding sorting and degradation, metabolism of cofactors and vitamins, metabolism of other amino acids, metabolism of terpenoids and polyketides, xenobiotics biodegradation and metabolism and endocrine system. However, the microbial community in sunny slope was mainly involved in signal transduction, cancers, xenobiotics biodegradation and metabolism, aging, biosynthesis of other secondary metabolites, energy metabolism, folding sorting and degradation, cell motility and translation.

**Fig. 6:**
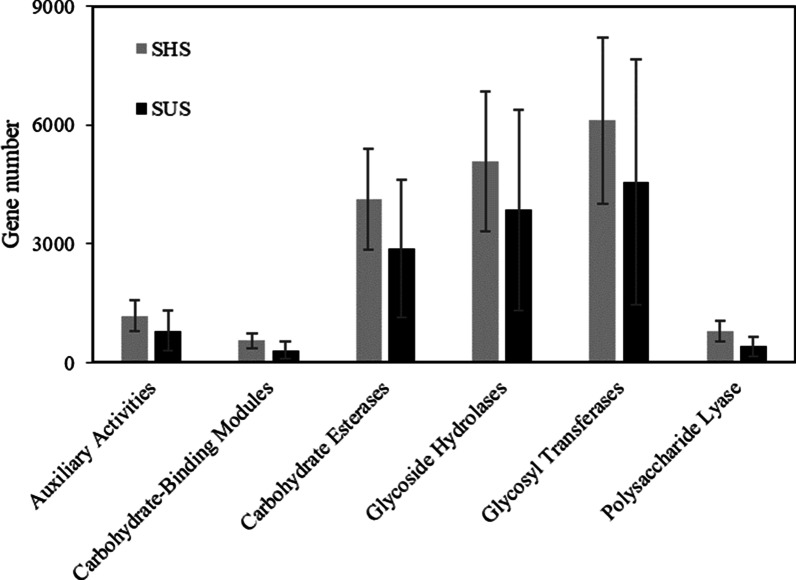
Variations of CAZy genes class number on sunny slope (SUS) and shady slopes (SHS)

Furthermore, microbial genes related to biomass conversion (CAZy genes) were annotated. CAZy (Carbohydrate-Active enzymes Database) is a professional database related to carbohydrate active enzymes, including related enzyme families that can catalyze carbohydrate degradation, modification and biosynthesis. CAZy contains six categories: Glycoside hydrolases (GHs), Glycosyltransferases (GTs), polysaccharide lyases (PLs), Carbohydrate esterases (CEs), Auxiliary activities (AAs) and Carbohydrate-Binding Modules (CBMs). As shown in Fig. 6, the gene number of CAZy in SHS were higher than that in SUS. In addition, the significant differences on CAZy families were mainly belonging to GHs and GTs (Additional file 1: Table S1).

### Relationships between different in microbial community composition and soil and plant variables

**Fig. 7:**
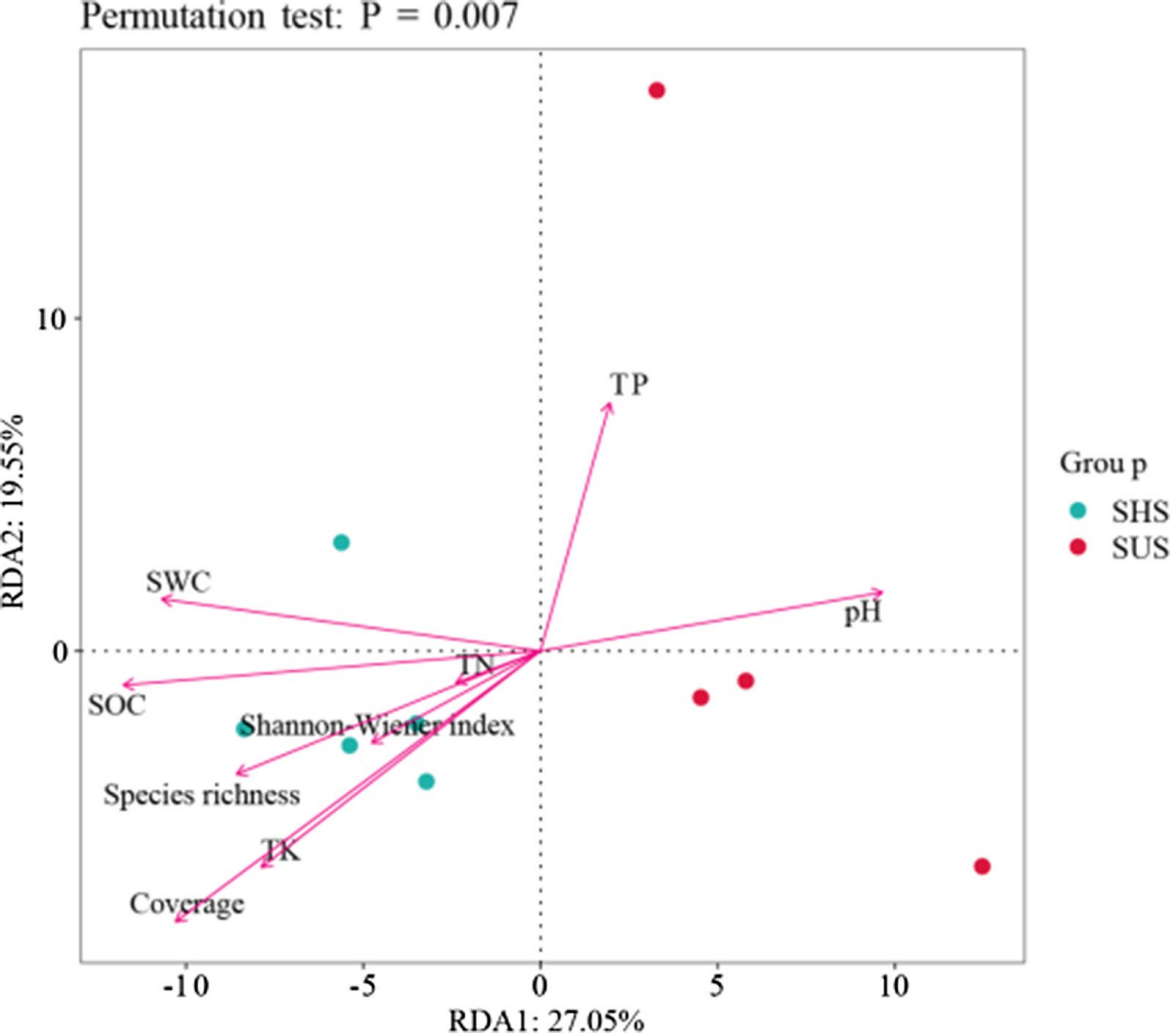
The redundancy analysis (RDA) of the soil characteristics or plant characteristics with microbial community on sunny slope (SUS) and shady slopes (SHS). *SWC* soil water content, *SOC* soil organic carbon, *TN* total nitrogen, *TP* total phosphorus, *TK* total potassium

The soil properties (SOC, TN, TP, TK, pH and SWC) and plant characteristics (Species richness, Cover and Shannon–Wiener index) were selected as environmental variables for microbial community (Fig. 7). RDA results showed that they could explain 46.6% of the microbial community composition variation. SOC, pH, SWC, species richness and coverage were the most key environmental variables influencing the composition of microbial community (explained for 74.9%, 62.15%, 68.4%, 59.2% and 83.2%, respectively). Based on Spearman correlation coefficient, significant correlations were also found between soil or plant characteristics and microbial community (Additional file 1: Fig. S7).

## Discussion

### Effects of slope aspect on vegetation characteristics and soil physicochemical properties

The inverted rock slope of the tiankeng serves as a bridge connecting the inside and the outside of the tiankeng, providing a breeding ground and seed propagation path for the plant community [23]. Studies have shown that slope aspect changes are an important cause of habitat heterogeneity. Accompanying differences in environmental factors such as light radiation, light duration, temperature, soil moisture and soil nutrients, which in turn affect the changes in the species composition and diversity of plant communities [24, 25]. Compared with the sunny slope, the shady slope has rich habitat resources and higher vegetation richness [26]. The deep sinking pond has a depth of 148.7 m and the west is a vertical cliff. The shaded slope is affected by the slope direction and the west vertical wall. The direct sunlight time is shorter than that of the sunny slope, and the amount of solar radiation is less than that of the sunny slope, which affects the vegetation distributed in the tiankeng. Our results founded that the dominant species on the shady slope were *Myrsine africana* Linn., *Debregeasia orientalis* C. J. Chen, *Ternstroemia gymnanthera* (Wight et Arn.) Beddome and the dominant species on the sunny slope were *Quercus guyavifolia*, *Quercus variabilis* Bl. Every plant in the community does not exist in isolation, but is the result of interdependence and co-evolution with the species that make up the community in the habitat, and is affected by the habitat [27]. The vegetation species richness and coverage are significantly higher on the shady slope. Studies have shown that species diversity increases with soil moisture [28]. The soil moisture on the sunny slopes has a large amount of evaporation, and the soil layer is shallow and discontinuous due to rocky desertification, and the water is easy to lose, resulting in low soil moisture. In addition, SOC, TN and TK all show that the shade slope is higher than the sunny slope, which is consistent with Liu et al. studies [29]. In general, the shady slopes in the karst tiankeng have better vegetation coverage and soil nutrition.

### Effects of slope aspect on microbial community structure and function

In the present study, the microbial community structure was different between the sunny slope and shady slope. Microbial community diversity was significantly higher in shady slope, which may due to the shady slope have suitable habitat for microbial community [30]. Suitable habitats mean higher resource levels, which allow more microbial species to meet their minimum resource requirements [31]. Carletti et al. also found that soil microbial communities in sunny and shady slope were significantly different [32]. Many studies have shown that shady slopes are conducive to nutrient accumulation, decomposition and microbial activity, which is consistent with our study [33, 34]. The PCoA analysis results also revealed a clear separation of microbial communities in different slope aspect (Fig. 2). The composition of the microbial community is similar on different slopes, but exhibits differences in microbial abundance. At the phylum level, *Proteobacteria* had the highest abundance in all soil samples, which were consistent with previous studies [5, 13]. In addition, *Actinobacteria, Chloroflexi, Acidobacteria, Verrucomicrobia* and *Firmicutes* were also abundant in all soil samples. *Proteobacteria, Actinobacteria, Chloroflexi* and *Acidobacteria* play a key role in organic matter transformation and nutrient cycle, which may suggested that microbes perform the strong metabolic capacity and nutrient degradation survive well in karst tiankeng [35]. At the genus level, *Bradyrhizobium*, *Streptomyces*, *Acidobacteria_noname* and *Betaproteobacteria_noname*. *Bradyrhizobium* have been regarded as dominant genera in soil [36]*.* The similar composition of the dominant genera indicates that tiankeng has a stable microbial community composition structure. However, the abundance of shared genera in different slope aspect is different, indicating that microorganisms with special functions can survive in different habitats in tiankeng. Therefore, different slope aspect of tiankeng has led to changes in the composition and structure of the microbial community. Furthermore, the most biomarkers of shady slope were belongs to *Actinobacteria* and *Chloroflexi.* However, the most biomarker of sunny slope was belongs to *Proteobacteria.* These results indicated that *Actinobacteria*, *Chloroflexi* and *Proteobacteria* were well adapted to the habitat in the karst tiankeng.

Based on metagenomic data, the metabolic potentials of microbiomes can be effectively analyzed. In this study, the microbes were main involved in metabolic pathways including carbohydrate metabolism, amino acid metabolism, energy metabolism. These pathways are essential for maintaining soil nutrient cycling and transformation [37]. The LDA results showed that the pathways biomarkers of shady slope were metabolism of other amino acids and xenobiotics biodegradation and metabolism. The pathways biomarkers of sunny slope were cell motility, translation and cell growth and death. The phenomenon that SUS has a higher abundance of genes associated with C and N cycles can be effectively explained by theory “nutrient limitation theory” [38]. SUS has relatively poor soil nutrients, making the growth of soil microorganisms more susceptible to nutrient conditions. Therefore, soil microbes maintain community construction by changing survival strategies, that is, increasing abundance of C and N cycles. This result also reflects the stabilization of nutritional status in SHS. It is worth noting that the microbial community on the sunny slopes were involved in human diseases, including infectious diseases, endocrine and metabolic diseases, cancers, cardiovascular diseases and drug resistance. Soil-borne pathogens that cause human disease constitute a minority of soil-dwelling species. Some soil-borne pathogens (such as bacterial genera *Pseudomonas* and *Enterobacter*) are opportunistic species that can infect humans. Although the potential of soil microbes in maintaining human health is less clear; rather, numerous studies confirm that soil biodiversity can bring enormous benefits. It is noteworthy that biodiversity loss in karst areas may have negative impacts on human health. Moreover, the abundance of CAZy genes was more abundant in shady slope. Auxiliary activities and glycoside hydrolases are considered related to the decomposition of litter [39]. The higher abundance of Auxiliary activities and glycoside hydrolases indicated that microorganisms from shady slope soil had stronger capability in litter decomposition. More abundant vegetation cover on shady slopes promotes nutrient cycling by producing more litter, which causes an increase in the abundance of CAZy genes. In general, the change in slope aspect affects the structure and function of the microbial community.

### Relationship among vegetation characteristics, soil properties and microbial community

Microbial communities are sensitive indicators of vegetation and soil properties changes, and their interaction is considered to be the main driving factor of ecosystem functions [40]. Many studies have indicated that the difference in soil microbial community structure is mainly affected by the vegetation characteristics and soil properties [22, 41]. The type of vegetation community determines the initial composition of the microbial community, and vegetation affects the structure of the microbial community by affecting the soil environment [42, 43]. The SOC content in karst areas is severely affected by soil erosion and degradation [44]. The higher SOC content in shady slope may have affected the microbial communities. High organic carbon is generally beneficial for rapid microbial growth [45]. pH has been widely regarded as an important soil properties affecting microbial communities in karst areas [5, 46]. Therefore, the difference in plant community composition and soil nutrients between shaded and sunny slopes is the main driving factor for the formation and evolution of microbial communities.

## Conclusions

This study reveals the microbial community structure and metabolic potentials characteristics of different slopes (shady and sunny slope) of karst tiankeng, which will help to deepen the understanding of the microbial diversity of karst tiankeng. The microbial communities Shannon–Wiener index was significantly higher in shady slope, and the microbial community structure differed between shady and sunny slope. The composition of dominant phyla at different slope aspect presented similarly but different abundances, and the dominant phyla were *Proteobacteria*, *Actinobacteria*, *Chloroflexi* and *Acidobacteria*. The main metabolic potentials pathways belong to metabolism and environmental information processing. LEfSe results indicated several biomarker pathways in sunny slope involved in human disease. Moreover, the abundance of CAZy genes was higher in shady slope and had stronger ability in decomposing litter. The microbial community is mainly related to vegetation species richness, coverage, SOC and pH.

## Methods

### Study area

The Zhanyi tiankeng group is located in Qujing County, Yunnan Province, China (25°35′–25°57′ N, 103°29′–103°39′ E). The Shenxiantang tiankeng is a representative degraded tiankeng in the Zhanyi tiankeng group (Additional file 1: Fig. S8). The area belongs to the subtropical plateau monsoon climate. Average annual temperature is 13.8 ~ 14 ℃, the annual rainfall is 1073.5 ~ 1089.7 mm, the annual total solar radiation energy is 123.8 kcal cm^−2^, and the average annual wind speed is 2.7 m s^−1^. The rocks in the area are dominated by carbonatite and dolomite, and the soil in the horizontal zone of yunnan red soil. The characteristics of Shenxiantang tiankeng have been reported in our previous research [23].

### Plant measurements and soil collection

In July of 2019, five and four different slope sites with an area of 20 × 20 m were selected in shady and sunny slope, respectively. In each site, three subplots were set along the diagonal, with an area of 1 m × 1 m were selected for the soil sampling. The plant coverage and dominant species were recorded. The plant community survey was only identified by observation and photographs, and comply with the Convention on the Trade in Endangered Species of Wild Fauna and Flora. The soil sample is collected from shenxiantang tiankeng and no need any permission. After removing surface weeds, soil samples were collected according to an “S” shape, and five points (0–10 cm) of soil were mixed to form a pooled soil sample after the large pieces of debris were removed. The fresh soil samples were stored in a plastic bag and transported to the laboratory. Parts of fresh soil sample were sieved (< 2 mm) and air dried for characterizing the soil physical and chemical properties [47]. Parts of fresh soil sample were used for DNA extraction.

### DNA extraction and sequencing

Microbial genomic DNA was extracted from 0.2 g fresh soil using the E.Z.N.A.® Soil DNA Kit (OMEGA, USA). The DNA quality and DNA concentration were assessed by agarose gel (1%, w/v) electrophoresis and Qubit 2.0 fluorometer (Life Technologies). After genomic DNA quality inspection, the genomic DNA was cut into DNA fragments (~ 500 bp) using the Covaris S220 (Covaris Inc., Woburn, MA, USA). The library was constructed by using NEB Next® Ultra™ DNA Library Prep Kit ((Illumina, San Diego, CA, United States)) according to the manufacture’s protocol. Paired-end sequencing was performed by PE150 (Illumina Inc., San Diego, CA, USA) at Sangon Biotech Co., Ltd (Shanghai, China).

### Processing of sequencing data

Remove raw reads and get clean reads was performed by using Trimmomatic [48]. The stitching software IDBA_UD was used to assemble the clean reads and get contig [49]. Each contig of the open reading frames (ORFs) were predicted by using the Prodigal, and ORFs (length ≥ 100 bp) were chosen and translated them into protein sequences [50]. Clustering 95% sequence identity of the gene sequences catalog and construct a nonredundant gene catalog were performed using CD-HIT (version 4.6) [51]. The Bowtie2 (version 2.1.0) and Samtools were used to get the abundance of gene in the sample [52]. DIAMOND (version 0.8.20) was used to compare assembled unigenes with Nr (NCBI non-redundant protein sequences) database for blastp homology to obtain functional annotation and homologous species information [53]. The KO numbers and pathways annotation information was obtained by compare protein sequence with KEGG database (Kyoto Encyclopedia of Genes and Genomes) using GhostKOALA (version 1.0) [54]. The carbohydrate enzymes active annotation by compare CAZy database (Carbohydrate-Active Enzymes) by using HMMER3 (version 3.1b1) [55].

### Data analysis

All of the statistical analyses were via SPSS software (version 22.0). Based on a Bray–Curtis matrix Principal, the coordinates analysis (PCoA) was calculated in QIIME (version 1.9.0). The Linear discriminant analysis effect size (LEfSe) was used to discover the potential microbial taxa and functional biomarkers (http://huttenhower.sph.harvard.edu/galaxy/root?tool_id=PICRUSt_normalize). The multivariate redundancy analysis (RDA) was performed in Canoco (version 5.0). The analysis of similarities (ANOSIM) was finished in the R vegan package. ANOVA analyses of differences in the microbial shannon diversity index, plant communities feature and soil physicochemical properties. The significance level was detected at the *p* < 0.05 and applied for all comparisons.

## Supplementary Information

**Additional file 1: Table S1.** The gene number of CAZy class on the different slopes. **Figure S1.** The Shannon-Wiener index of microbial community on sunny slope (SUS) and shady slopes (SHS). The different letters mean significant difference at *p* < 0.05. **Figure S2.** The analysis of similarities (AMOSIM) of microbial community on sunny slope (SUS) and shady slopes (SHS). **Figure S3.** The abundance of microbial community composition on sunny slope (SUS) and shady slopes (SHS). **Figure S4.** The LEfSe analysis of microbial community composition on sunny slope (SUS) and shady slopes (SHS) (threshold value of 3.0). **Figure S5.** The microbial community function pathways on sunny slope (SUS) and shady slopes (SHS). **Figure S6.** The abundance of genes associated with C and N cycle on sunny slope (SUS) and shady slopes (SHS). **Figure S7.** The correlation between the microbial community (at phylum level) and environmental variables. * correlation significant at the 0.05 level. SWC, soil water content; SOC, soil organic carbon; TN, total nitrogen; TP, total phosphorus; TK, total potassium. **Figure S8.** Location of the study site in Yunnan Province, China (The data set is provided by Geospatial Data Cloud site, Computer Network Information Center, Chinese Academy of Sciences. (http://www.gscloud.cn)).

## Data Availability

The sequences were submitted to the SRA at NCBI under the accession number PRJNA814514.

## Abbreviations

SWC: Soil water content
SOC: Soil organic carbon
TN: Total nitrogen
TP: Total phosphorus
TK: Total potassium
