# Supplementary material for: Slope aspect affects the soil microbial communities in karst tiankeng negative landforms
Source: BMC Ecol Evol. 2022 Apr 30;22:54. doi: 10.1186/s12862-022-01986-y (PMC9063220; doi:10.1186/s12862-022-01986-y)
Supplement: Supplementary file 1 — Additional file 1: Table S1. The gene number of CAZy class on the different slopes. Figure S1. The Shannon-Wiener index of microbial community on sunny slope (SUS) and shady slopes (SHS). The different letters mean significant difference at p < 0.05. Figure S2. The analysis of similarities (AMOSIM) of microbial community on sunny slope (SUS) and shady slopes (SHS). Figure S3. The abundance of microbial community composition on sunny slope (SUS) and shady slopes (SHS). Figure S4. The LEfSe analysis of microbial community composition on sunny slope (SUS) and shady slopes (SHS) (threshold value of 3.0). Figure S5. The microbial community function pathways on sunny slope (SUS) and shady slopes (SHS). Figure S6. The abundance of genes associated with C and N cycle on sunny slope (SUS) and shady slopes (SHS). Figure S7. The correlation between the microbial community (at phylum level) and environmental variables. * correlation significant at the 0.05 level. SWC, soil water content; SOC, soil organic carbon; TN, total nitrogen; TP, total phosphorus; TK, total potassium. Figure S8. Location of the study site in Yunnan Province, China (The data set is provided by Geospatial Data Cloud site, Computer Network Information Center, Chinese Academy of Sciences. (http://www.gscloud.cn)). [file 12862_2022_1986_MOESM1_ESM.docx]

**Additional materials**

**Table S1.** The gene number of CAZy class on the different slopes.

| **CAZy Class** | **CAZy Abbr** | **SHS** | **SUS** | **P value** |
| --- | --- | --- | --- | --- |
| Auxiliary Activities | AA5 | 0.1396 | 0.1849 | 0.018 |
| Carbohydrate-Binding Modules | CBM12 | 0.0745 | 0.0980 | 0 |
|  | CBM38 | 0.0265 | 0.0186 | 0.028 |
|  | CBM40 | 0.0223 | 0.0279 | 0.011 |
|  | CBM41 | 0.0240 | 0.0337 | 0.05 |
|  | CBM51 | 0.0182 | 0.0137 | 0.02 |
|  | CBM61 | 0.0195 | 0.0145 | 0.015 |
| Carbohydrate Esterases | CE1 | 0.0147 | 0.0074 | 0.001 |
|  | CE15 | 0.0111 | 0.0139 | 0.005 |
|  | CE16 | 0.0108 | 0.0195 | 0.006 |
|  | CE2 | 0.0157 | 0.0104 | 0.016 |
|  | CE3 | 0.0117 | 0.0077 | 0 |
| Glycoside Hydrolases | GH100 | 0.0109 | 0.0074 | 0.027 |
|  | GH104 | 0.0063 | 0.0036 | 0.017 |
|  | GH119 | 0.0083 | 0.0040 | 0.011 |
|  | GH12 | 0.0071 | 0.0047 | 0.042 |
|  | GH13 | 0.0041 | 0.0024 | 0.012 |
|  | GH17 | 0.0030 | 0.0013 | 0.005 |
|  | GH19 | 0.0025 | 0.0052 | 0.014 |
|  | GH2 | 0.0031 | 0.0020 | 0.04 |
|  | GH25 | 0.0054 | 0.0017 | 0.002 |
|  | GH29 | 0.0021 | 0.0013 | 0.046 |
|  | GH30 | 0.0048 | 0.0020 | 0.018 |
|  | GH4 | 0.0025 | 0.0010 | 0.012 |
|  | GH46 | 0.0030 | 0.0017 | 0.043 |
|  | GH49 | 0.0023 | 0.0008 | 0.02 |
|  | GH50 | 0.0023 | 0.0005 | 0.001 |
|  | GH53 | 0.0016 | 0.0006 | 0.005 |
|  | GH54 | 0.0016 | 0.0011 | 0.038 |
|  | GH55 | 0.0016 | 0.0006 | 0 |
|  | GH57 | 0.0016 | 0.0010 | 0.021 |
|  | GH68 | 0.0014 | 0.0008 | 0.039 |
|  | GH7 | 0.0019 | 0.0004 | 0.009 |
|  | GH70 | 0.0011 | 0.0003 | 0.001 |
|  | GH75 | 0.0008 | 0.0004 | 0.028 |
|  | GH76 | 0.0008 | 0.0004 | 0.039 |
|  | GH78 | 0.0010 | 0.0006 | 0.022 |
|  | GH94 | 0.0010 | 0.0002 | 0.006 |
| Glycosyl Transferases | GT12 | 0.0007 | 0.0002 | 0.029 |
|  | GT14 | 0.0003 | 0.0000 | 0.002 |
|  | GT2 | 0.0005 | 0.0002 | 0.015 |
|  | GT21 | 0.0003 | 0.0001 | 0.01 |
|  | GT26 | 0.0010 | 0.0002 | 0.043 |
|  | GT30 | 0.0002 | 0.0007 | 0.014 |
|  | GT39 | 0.0003 | 0.0001 | 0.008 |
|  | GT40 | 0.0001 | 0.0000 | 0.003 |
|  | GT47 | 0.0005 | 0.0001 | 0.044 |
|  | GT58 | 0.0002 | 0.0000 | 0.018 |


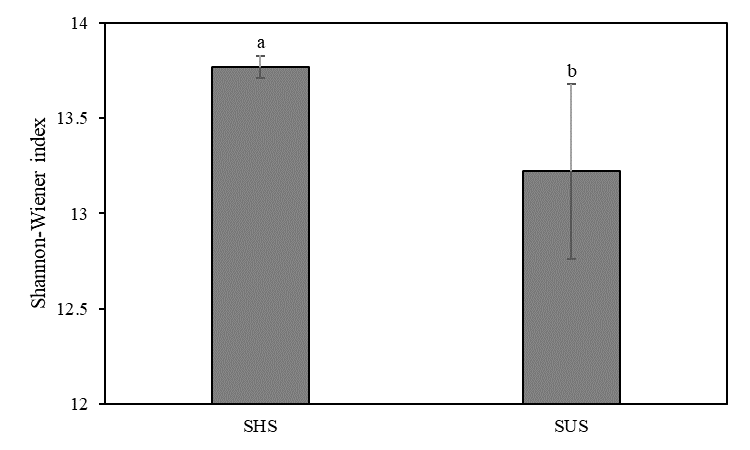


**Figure S1.** The Shannon-Wiener index of microbial community on sunny slope (SUS) and shady slopes (SHS). The different letters mean significant difference at *p* < 0.05.


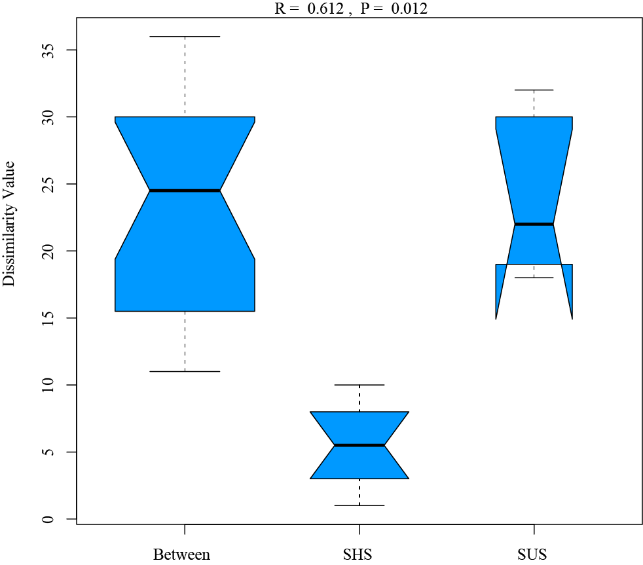


**Figure S2.** The analysis of similarities (AMOSIM) of microbial community on sunny slope (SUS) and shady slopes (SHS).


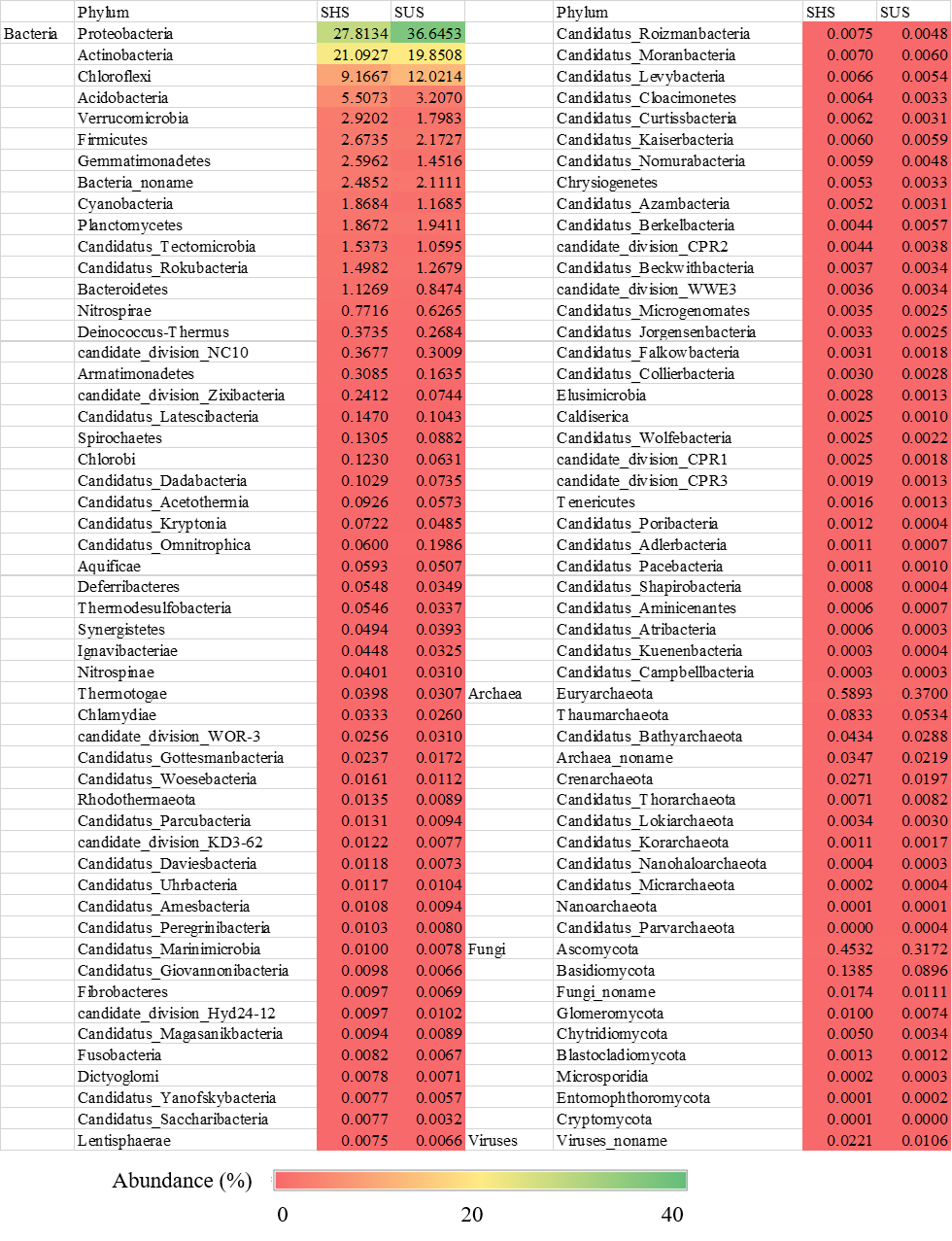


**Figure S3.** The abundance of microbial community composition on sunny slope (SUS) and shady slopes (SHS).


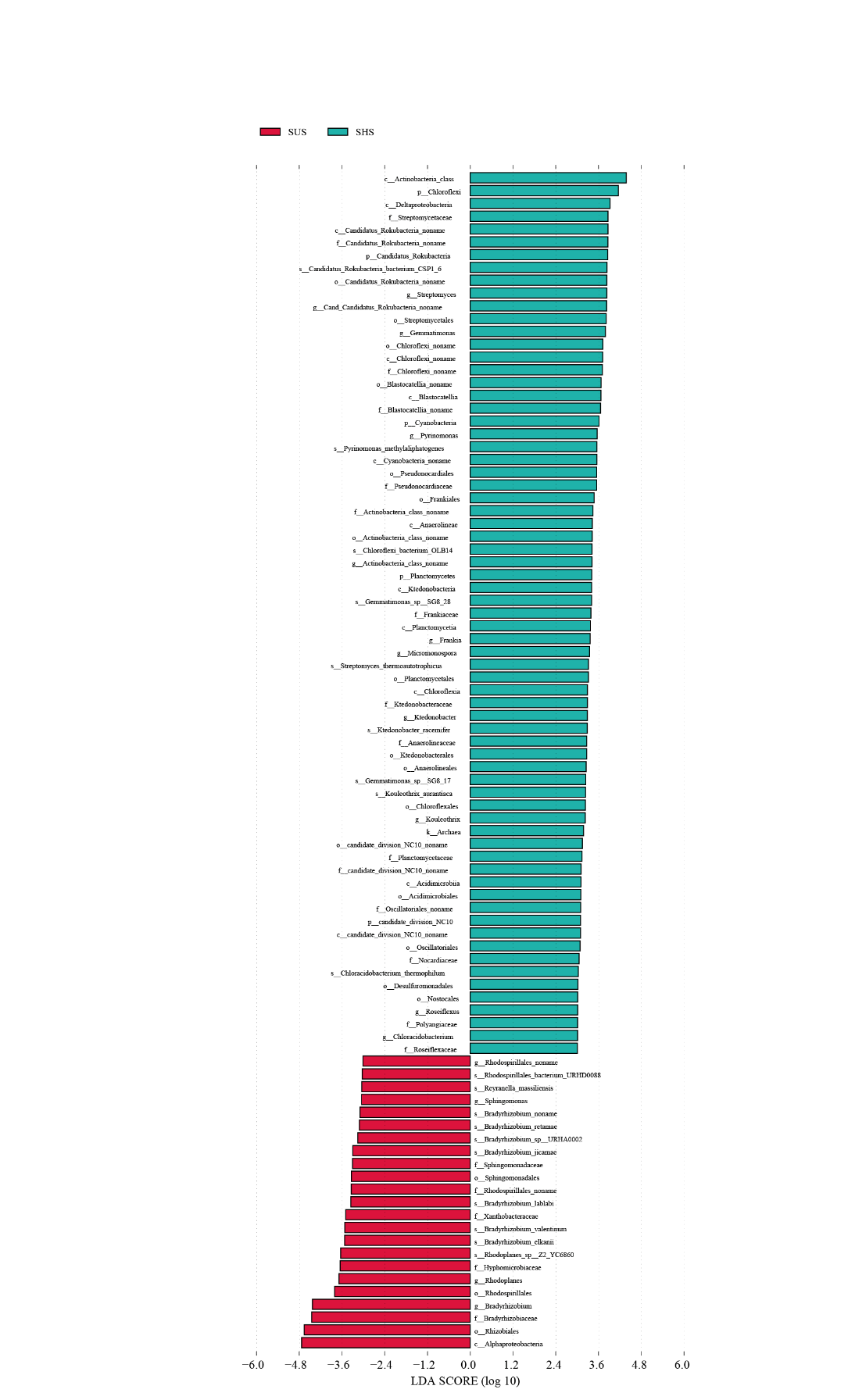


**Figure S4.** The LEfSe analysis of microbial community composition on sunny slope (SUS) and shady slopes (SHS) (threshold value of 3.0).


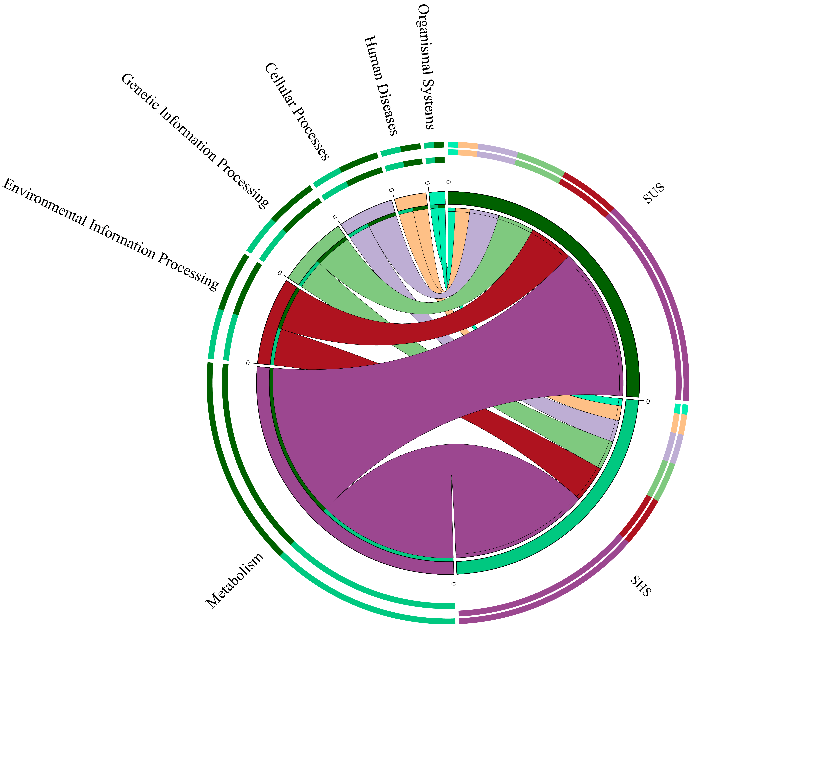


**Figure S5.** The microbial community function pathways on sunny slope (SUS) and shady slopes (SHS).


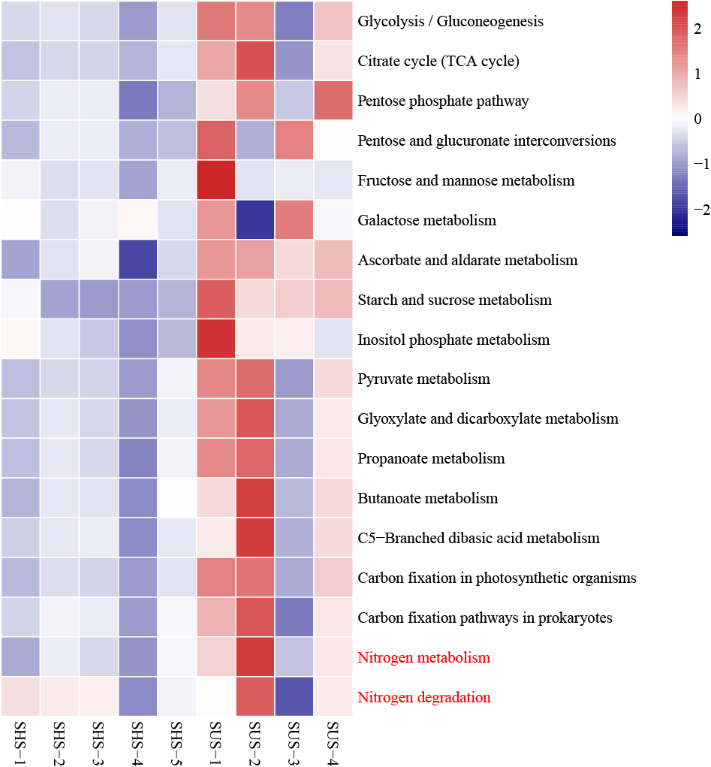


**Figure S6.** The abundance of genes associated with C and N cycle on sunny slope (SUS) and shady slopes (SHS).


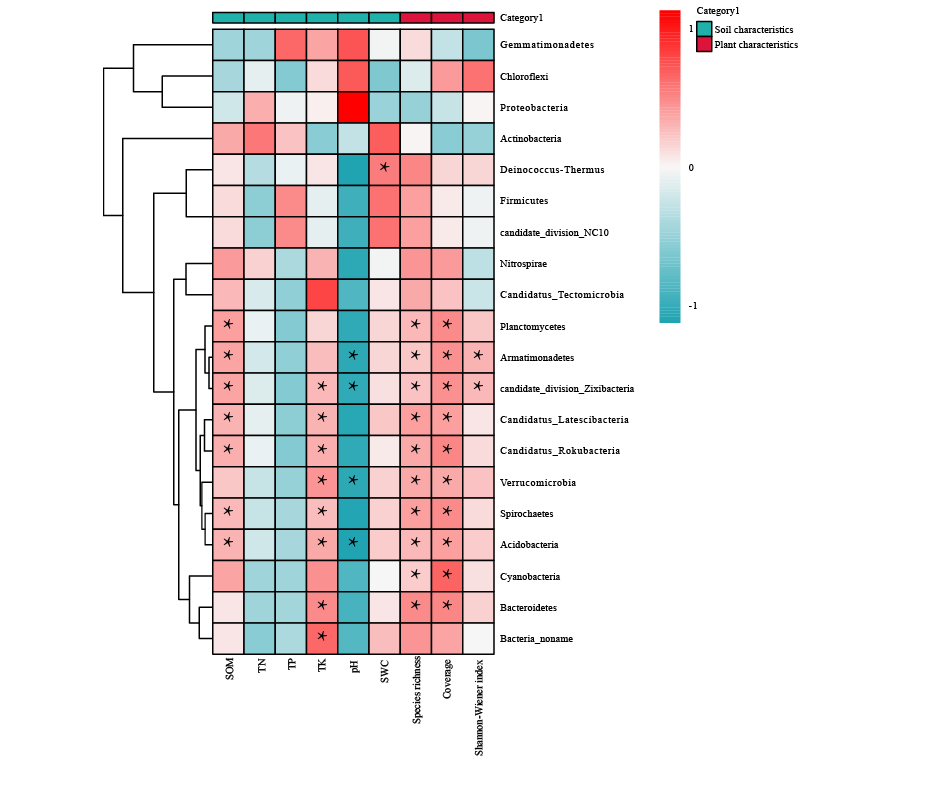


**Figure S7.** The correlation between the microbial community (at phylum level) and environmental variables. * correlation significant at the 0.05 level. SWC, soil water content; SOC, soil organic carbon; TN, total nitrogen; TP, total phosphorus; TK, total potassium.


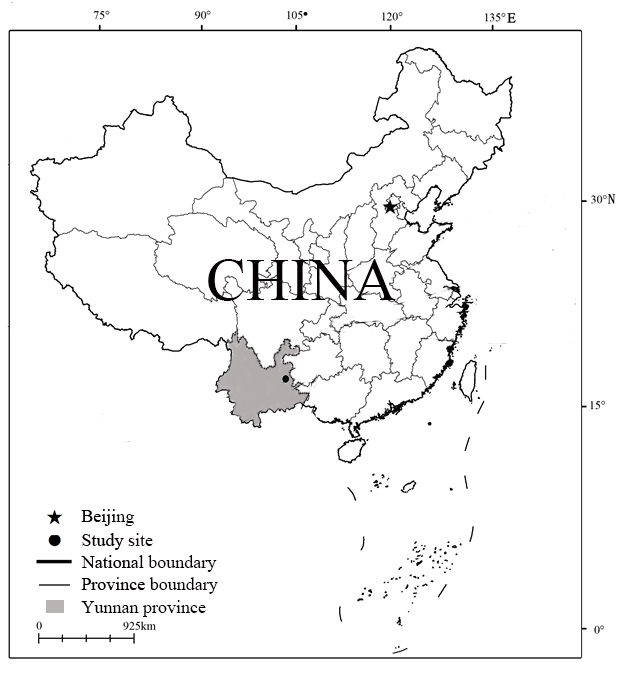


**Figure S8.** Location of the study site in Yunnan Province, China (The data set is provided by Geospatial Data Cloud site, Computer Network Information Center, Chinese Academy of Sciences. (http://www.gscloud.cn)).
